# Mediators of Mast Cells in Bullous Pemphigoid and Dermatitis Herpetiformis

**DOI:** 10.1155/2014/936545

**Published:** 2014-10-21

**Authors:** Agnieszka Zebrowska, Malgorzata Wagrowska-Danilewicz, Marian Danilewicz, Olga Stasikowska-Kanicka, Lilianna Kulczycka-Siennicka, Anna Wozniacka, Elzbieta Waszczykowska

**Affiliations:** ^1^Department of Dermatology and Venereology, Medical University of Lodz, Plac Hallera 1, 90-497 Lodz, Poland; ^2^Laboratory of Nephropathology of Medical University of Lodz, Pomorska 251, 92-213 Lodz, Poland

## Abstract

Bullous pemphigoid (BP) and dermatitis herpetiformis (DH) are skin diseases associated with inflammation. However, few findings exist concerning the role of mast cells in autoimmune blistering disease. Skin biopsies were taken from 27 BP and 14 DH patients, as well as 20 healthy individuals. Immunohistochemistry was used to identify the localization and mast cell expression of TNF*α* and MMP9 in skin lesions and perilesional skin. The serum concentrations of TNF*α*, MMP9, chymase, tryptase, PAF, and IL-4 were measured by immunoassay. TNF*α* and MMP9 expression in the epidermis and in inflammatory influxed cells in the dermis was detected in skin biopsies from patients. Although these mediators were found to be expressed in the perilesional skin of all patients, the level was much lower than that in lesional skin. Increased serum PAF levels were observed in BP patients. Mast cells may play an essential role in activating inflammation, which ultimately contributes to the tissue damage observed in BP and DH. Our findings suggest that differences in the pattern of cytokine expression directly contribute to variations in cellular infiltration in DH and BP.

## 1. Introduction

Dermatitis herpetiformis (DH) is one of the subepidermal autoimmune bullous diseases, which is characterized by skin and intestinal lesions. Skin lesions include polymorphic eruption accompanied by severe pruritus. Intestinal lesions are characterized by atrophy of intestinal villi resulting from immunological process [1]. Diagnosis of DH is established on the basis of a direct immunofluorescence test (DIF) revealing granular deposits of IgA in the papillae and the presence of circulating IgA antibodies directed against the endomysium and/or tissue and epidermal transglutaminase (tTG, eTG) [2, 3]. Skin lesions in DH are histologically characterized by neutrophilic infiltrates leading to destruction of basement membrane zone (BMZ) proteins and anchoring fibers and blister formation [4–6].

Bullous pemphigoid (BP) is a blistering disease characterized by inflammatory infiltrate in the dermis and the presence of IgG and C3 deposits along the BMZ and circulating IgG autoantibodies. Autoantibodies bound to autoantigens, the glycoproteins BPAG1 (230 kD) and BPAG2 (180 kD), localized in the basement membrane of the epidermis activate a series of immunological and enzymatic phenomena leading to the destruction of basement membrane components and the formation of blisters, as observed in DH [7, 8].

In the dermis, inflammatory infiltrates formed by eosinophils and neutrophils and bound* in vivo* deposits, can be observed along the basement membrane or at the top of papillae. Ultrastructural studies have also confirmed the presence of intensive inflammatory infiltrates at the dermoepidermal junction, as well as destruction of hemidesmosomes and components of the extracellular matrix [9].

Infiltrate formation is preceded by early accumulation of leukocytes, depending on the activity of adhesion molecules. The binding of autoantibodies leads to the activation of keratinocytes, which release cytokines, as well as the activation of metalloproteinases and the C5 component of the complement [10, 11]. Many mediators are important chemoattractants for both eosinophils and neutrophils [12, 13].

Mast cells are a source of many mediators, cytokines, and enzymes which may affect the course of inflammation in the skin in different ways. One mediator which plays a very important role in the development skin lesions in autoimmune skin diseases is tumor necrosis factor *α* (TNF*α*). Other cytokines derived from mast cells also appear to be involved in the inflammatory process in skin. In the course of BP and DH, an important role is played by mast cell mediators such as histamine, tryptase, and chymase. Evidence suggests that metalloproteinase (MMP9 in particular), leukotrienes (LT), platelet activating factor (PAF), and heparin derived from mast cells play a role in the inflammatory process involved in blister formation [14]. Recent data on the role of cytokines and mediators from mast cells in blister formation should be taken into account in the planning of new methods for treating these diseases. The aim of the study was to evaluate the expression of these markers of mast cells in skin lesions and the perilesional area, as well as in the serum of patients with DH and BP.

## 2. Materials and Methods

### 2.1. Patients

The study included 61 persons: 27 untreated patients with BP (range: 58 to 84 years, average: 68.5) and 14 with DH (range: 18 to 70 years, average: 49.8) in an active stage of the disease. The control group comprised 20 healthy individuals in total. The mean age of control group number 1 (10 patients), for BP patients, was 71.6 years (range 50 to 80 years) while the mean age of control group number 2 (10 patients), DH patients, was 42 years (range 19 to 49 years). The control groups consisted of unrelated volunteers matched for sex and age.

In all DH cases, histological examination revealed perivascular neutrophilic infiltrates, the presence of Pierrard's abscesses, and small subepidermal blisters. In most samples, large unilocular blisters displaying multiple neutrophilic papillary microabscesses were found. All histopathological findings according to Ackerman were fully developed [15]. Direct immunofluorescence tests revealed the presence of granular deposits of IgA in skin papillae and all indirect immunofluorescence tests were positive for IgAEmA (Oesophagus monkey IgAEmA, Medizinische Labordiagnostica). Immunoassay (Celikey, Pharmacia & Upjohn) revealed the presence of anti-tissue transglutaminase antibodies in 8/14 cases. Diagnosis of DH was established based on clinical presentation and results of histological and immunological examination.

Twenty-seven patients (16 women, 11 men; mean age 68.5 years; range: 58–84) with BP were included in the study. Pemphigoid was diagnosed based on clinical picture and histological and immunological findings. The patients were at an active stage of the disease; 22 of the 27 patients presented with skin blisters, vesicles, and itching papules, whereas the rest had only small vesicles and urticarial papules. In all cases, the histopathology findings were fully developed according to Ackerman et al. [15]. In all patients, direct immunofluorescence assay revealed IgG/C3 linear deposits along the BMZ. In the salt split test, deposits were observed in the epidermal part of the blister. Indirect immunofluorescence assay revealed circulating IgG antibodies to be present in all patients. According to ELISA, anti-Nc16 autoantibodies (MBL, Nagoya, Japan) were present in the serum of 21 out of 27 patients. The clinical diagnosis was supported by typical histological features of BP, including the presence of neutrophilic infiltrates, eosinophils, and lymphocytes (as well as subepidermal blisters in 22 cases).

All the patients gave their informed written consent before entering the study. The study protocol (RNN/132/07/KB) was approved by the Local Ethical Committee of the Medical University of Lodz.

*Tissue Specimens.* The biopsies were taken from the skin of the buttock or trunk before administration of any (topical or systemic) treatment. Skin lesions lasted between 2 weeks and 4 months. Biopsy specimens were taken from skin of the buttock from healthy volunteers.

### 2.2. Immunohistochemistry

Paraffin-embedded sections, 3-4 *μ*m thick, were used for routine H&E staining and for immunoperoxidase immunohistochemical examination with the DAKO EnVision detection system. The following primary monoclonal antibodies were used: anti-TNF*α* (R&D, UK) and matrix metalloproteinase 9 (MMP9) (Novocastra).

For immunohistochemistry, the paraffin-embedded sections were placed on adhesive plates and dried at 56°C for 24 hours and were later deparaffinated in a series of xylenes and alcohols with decreasing concentrations. The activity of endogenous peroxidase was inhibited with 3% hydrogen peroxide solution in methanol for 5 minutes.

In order to retrieve the antigenicity of tissues and allow them to react with antibodies, specific procedures were used for each tested antibody, according to the manufacturer's instructions. After incubation with diluted antibodies for 60 minutes at room temperature, they were washed with Tris buffer twice. DAKO EnVision double-step visualization system was then applied in order to visualize the antigen-antibody reaction. In the case of a positive immunohistochemical reaction, cellular nuclei were stained with Meyer haematoxylin for 2 minutes. After dehydration and processing through a series of acetones and xylenes, the sections were fixed in Canadian balm.

### 2.3. Semiquantitative Analysis

In each specimen, the staining intensity of MMP9 and TNF*α* in inflammatory infiltrates was recorded by two independent observers in 7–10 adjacent high power fields. They were graded from 0 (staining not detectable), 1 (minimal immunostaining in some cells), 2 (weak immunostaining intensity in all cells), and 3 (strong staining in all cells). The mean grade was calculated by averaging the grades assigned by the two authors and approximating the arithmetical mean to the nearest unity.

### 2.4. Morphometry

Histological morphometry of MMP9 and TNF*α* immunoexpression by epithelial and inflammatory cells was performed by using an image analysis system. The hardware comprised a PC with Indeo Fast card (frame grabber, true colour, real time) (Indeo, Taiwan) and colour video camera (Panasonic, Japan) linked to a Jenaval microscope (Carl Zeiss, Germany). The software used was MultiScan 8.08 (Computer Scanning Systems, Poland). The following values were calculated: the number of objects (semiautomatic function) and the surface area of the structure based on a stereological grid, with a regulated number of points.

The percentage of immunopositive inflammatory cells was estimated by counting 100–120 inflammatory cells in 7–10 adjacent high power fields on each slide using the semiautomatic function. The staining in the epithelial cells was measured using the point-counting method, based on Weibel [16]. The point spacing was 16 *μ*m. The total number of points in the grid was 169, and total area was 36864 sq. *μ*m. Using this grid, 7–10 randomly selected adjacent fields of the epithelium were investigated. The percentage of positive staining was calculated as the percentage of the number of points overlying positive areas with regard to the total number of points counted.

### 2.5. Statistical Methods

Median levels of TNF and MMP9 for patients with BP and DH in lesions (L) and surrounding (S) were compared using the nonparametric Mann-Whitney* U* test. Results were considered statistically significant if *P* < 0.05.

### 2.6. Serum Chemokine Levels

In order to determine the concentrations of the examined protein in the serum, the enzyme-linked immunoassay method was used: chymase: ELISA Kit for Human Chymase 1 Mast Cell (Uscn Life Science), tryptase: ELISA Kit for Human Tryptase (Uscn Life Science), TNF*α*: Human TNF*α* ELISA Kit (Gen-Probe Diaclone), IL-4: Human IL-4 ELISA Kit (Gen-Probe Diaclone), PAF: ELISA Kit for Human Platelet Activating Factor (PAF) (Uscn Life Science), and MMP9 (Quantikine, R&D Systems).

Chymase, tryptase, TNF*α*, PAF, MMP9, and IL-4 levels were measured in serum in all patients and healthy controls undergoing skin biopsy. Samples of 5cc venous blood were drawn from the ulnar vein, and, after centrifugation, the serum was stored at −20°C for an immunoassay.

### 2.7. Statistical Methods

All data was presented as median and range. Nonparametric Kruskal-Wallis analysis was performed followed by a median test. Significance at *P* < 0.05 was taken as statistically significant. Statistical analysis was carried out using Statistica 10 software (Statsoft Polska).

## 3. Results

### 3.1. Serum Chemokine Levels

**Figure 1:**
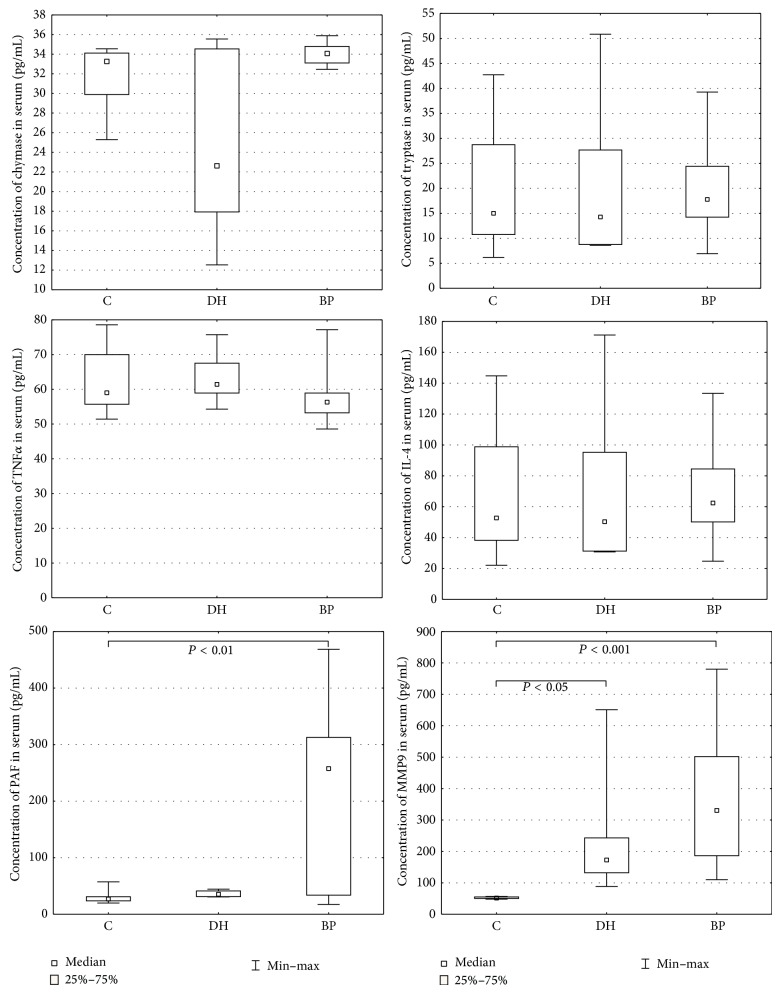
Serum levels of examined mediators in BP, DH, and control groups.

PAF levels were statistically higher in BP patients (218.19 +/− 29.874) than healthy subjects 29.87 +/− 2.973 (*P* = 0.004981). No differences were found between DH patients and the control group (*P* = 0.614416), Figure 1.

MMP9 levels were significantly higher in BP patients (364.79 +/− 41.383) and patients with dermatitis herpetiformis (243.10 +/− 77.110) compared to healthy subjects (52.61 +/− 0.779): *P* = 0.000002 and *P* = 0.029907, respectively, Figure 1.

The chymase (34.05 +/− 0.209 versus 24.30 +/− 3.438 versus 32.10 +/− 0.823), tryptase (19.79 +/− 1.983 versus 20.74 +/− 6.083 versus 19.33 +/− 3.231), IL-4 (68.66 +/− 6.653 versus 71.50 +/− 20.219 versus 66.98 +/− 10.850), and TNF*α* (57.26 +/− 1.263 versus 63.57 +/− 3.318 versus 62.11 +/− 2.558) levels were similar in BP and DH patients, as well as healthy controls, (Figure 1).

### 3.2. Expression of TNF*α* in Skin

**Figure 2:**
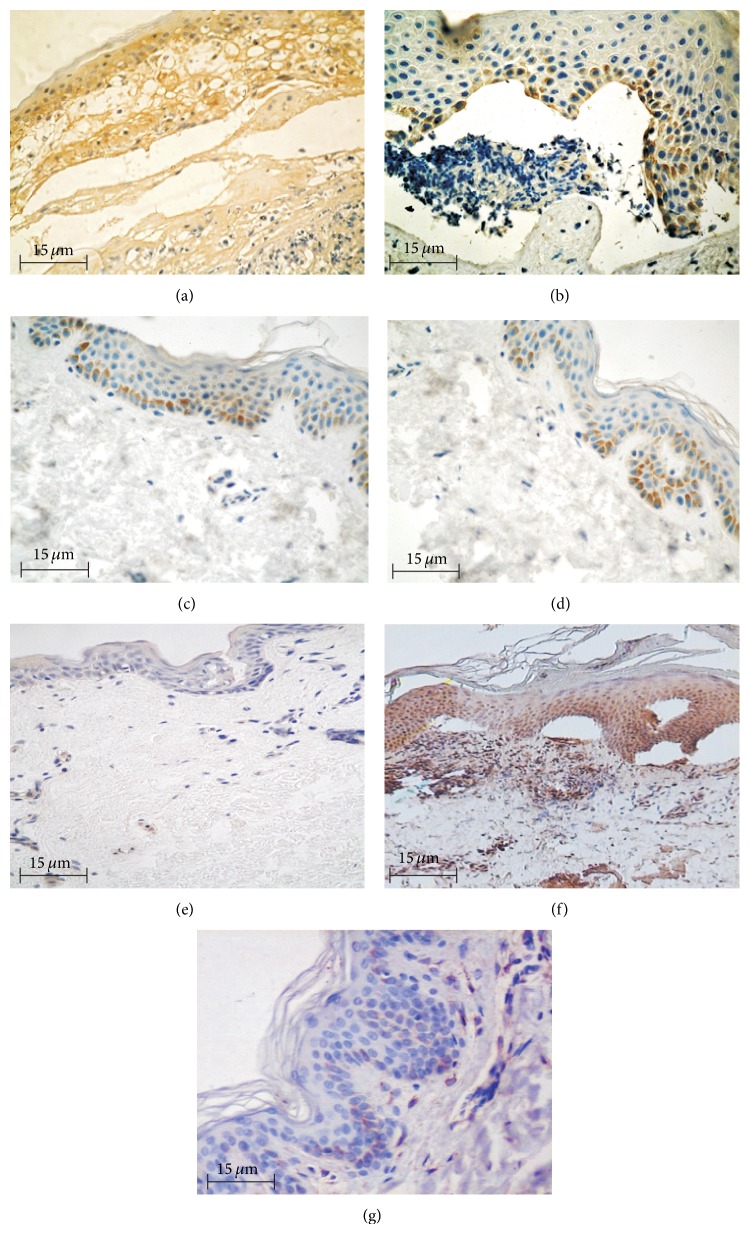
Immunoexpression of TNF*α* and MMP9 in epidermis and influx, 400x, immunohistochemistry. (a) Immunoexpression of TNF*α* in epidermis and influx, skin lesions (BP). (b) Immunoexpression of TNF*α* in epidermis, skin lesion (DH). (c) Immunoexpression of TNF*α* in epidermis, perilesional skin (BP). (d) Immunoexpression of TNF*α* in epidermis, perilesional skin (DH). (e) Negative immunoexpression of TNF*α*, normal skin. (f) Immunoexpression of MMP9 in epidermis and influx, skin lesions (BP). (g) Almost negative immunoexpression of MMP9, normal skin.

Moderate expression of TNF*α* was found in inflammation cells, the basal keratinocytes, and blister fluid (Figure 2). TNF*α* expression was also observed in BP (17/27) and DH (9/14) perilesional skin (Figure 2). Immunohistochemistry revealed TNF*α* expression to be absent in the skin biopsies from control patients (Figure 2).

**Figure 3:**
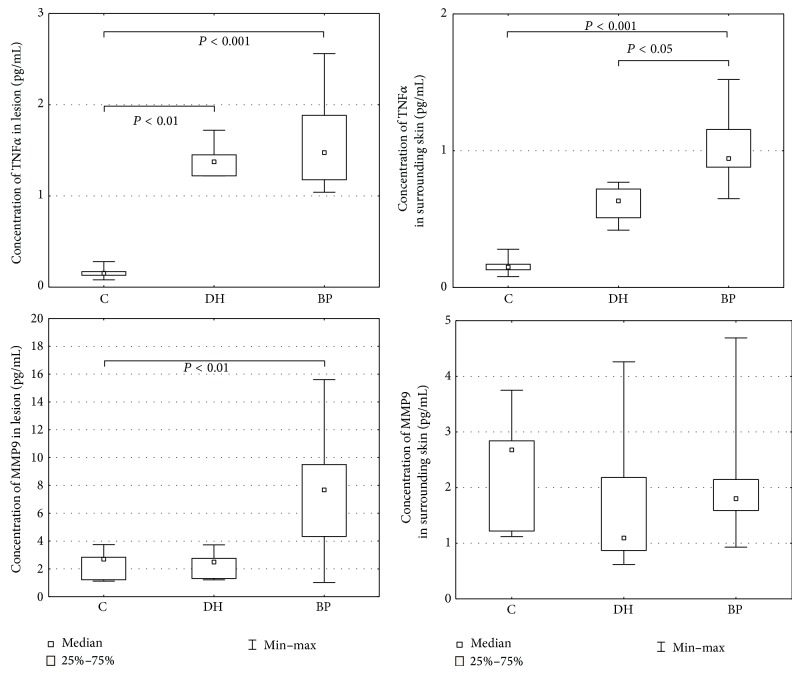
Morphometry of the immunoexpression of MMP9 and TNF*α*, skin lesion.

**Figure 4:**
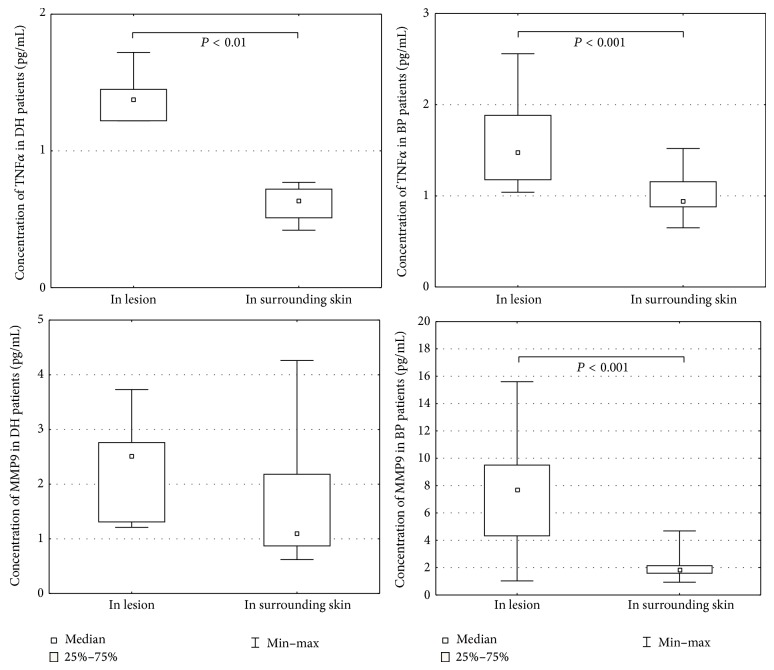
Morphometry of the immunoexpression of MMP9 and TNF*α*, perilesional skin.

Morphometric analysis revealed that TNF*α* expression was significantly higher in lesions than the surrounding skin: in BP 1.59 ± 0.100 versus 1.01 ± 0.045 (*P* = 0.000005) and DH 1.39 ± 0.069 versus 0.62 ± 0.050 (*P* = 0.004998) (Table 1) (Figure 3). Expression was significantly higher in BP and DH skin lesions versus control group (1.59 ± 0.100 versus 0.16 ± 0.017 and 1.39 ± 0.069 versus 0.16 ± 0.017, resp., *P* = 0.000004 and 0.007028) and in BP perilesional skin versus control group (1.01 ± 0.045 versus 0.16 ± 0.017, *P* = 0.0000001) (Figure 4).

### 3.3. Expression of MMP9 in Skin

Moderate expression of MMP9 was found in inflammation cells in BP patients, as well as in the epidermal basal cells in DH patients (Figure 2). MMP9 expression was also observed in BP and DH patients in perilesional skin. Immunohistochemistry found MMP9 expression to be absent in the skin biopsies from control patients (Figure 2).

Morphometric analysis revealed that MMP9 expression was significantly higher in lesions than the surrounding skin in patients with BP (7.41 ± 0.814 versus 2.01 ± 0.183, *P* = 0.000045) (Table 1) and in those with DH (2.34 ± 0.356 versus 1.69 ± 0.511). Expression was significantly higher in skin lesions in the BP group compared to the control group (7.41 ± 0.814 versus 2.15 ± 0.276). No differences were found between the expression of MMP9 in DH skin lesions versus perilesional skin (*P* = 0.128206) (Figures 3 and 4).

## 4. Discussion

D'Auria et al. [17] have studied the correlation between the enzymes myeloperoxidase, a product specific for granulocytes, and tryptase, a proteolytic enzyme synthesized and released by mast cells, with the levels of various cytokines in the blister fluid. They note a positive correlation between the levels of tryptase, IL-8, and RANTES, showing high activity of cytokines in relation to influx of eosinophils. A few studies confirm that eosinophils are very important cells in the formation of infiltration in BP patients. Markers of eosinophils and neutrophils have an important influence on the cytokine network, as well as other factors, such as adhesion molecules, tissue factors, and chemokines. The elevated expression of mast cell mediators in skin lesions and serum suggests that activation of these cells in bullous diseases is also important.

It seems important to determine which of the mediators secreted from mast cells are involved in the development of skin lesions in blistering diseases. Mast cell markers include tryptase, chymase, IL-4, PAF, TNF*α*, and the MMP9 enzyme.

As demonstrated by Heimbach et al. [18], the activation of complement and mast cell degranulation in a mouse model of BP is mediated by the C5a receptor. Recent studies confirm that these cells not only are involved in response to pathogens and in the mechanisms of allergy, but also are responsible for the development of neutrophilic infiltration [19].

Using an animal BP model, Chen et al. [20, 21] have also confirmed that mast cells are responsible for the influx of neutrophils and macrophages into tissues, which causes the destruction of the dermal-epidermal junction. Of the mast cell markers examined in the present study, significantly higher levels were found for PAF and MMP9 in patients with BP.

The mechanisms leading to mast cell activation and release of proteases in these diseases are poorly understood, but C3a and C5a anaphylatoxins, neuropeptides, and cytokines could be involved as well as numerous other proteins or peptides. The involvement of mast cells in inflammation and blister formation in subepidermal bullous diseases has been suggested, and since early skin lesions have an urticarial appearance, the mast cells are hypogranulated, and their granules are spread extracellularly and blister fluid contains increased histamine and tryptase levels. In addition, injection of the mast cell degranulator, compound 48/80, into the skin of patients with dermatitis herpetiformis causes a DH-like bullous lesion in some cases [22]. The results suggest that mast cell proteases participate in the inflammatory reaction; however, their direct role in blister formation is not clear. It is possible that PAF and TNF*α* induce and maintain the blisters by continuously activating collagenolytic metalloproteinases and degrading fibronectin.

The expression of TNF*α* in bullous lesions in both diseases was also significantly elevated compared to healthy subjects. The significantly reduced expression of this cytokine observed in normal looking skin was also important, suggesting the participation of TNF*α* in the formation of inflammatory influx and blisters.

Our findings confirm the role of TNF*α* and mast cells in the formation of skin lesions through the development of neutrophilic infiltration and the subsequent activation of immune mechanisms. Mediators secreted by these cells may influence the early changes which have been described, such as urticarial wheals accompanied by strong itching, in the course of a BP.

The destruction of tissue in the disorders associated with the increased activity of matrix metalloproteinases is often the result of an imbalance between the activity of these enzymes and their inhibitors. Recently, MMPs have been reported to play a significant role in the pathogenesis of skin diseases, including blistering diseases [23]. Enzymes are thought to play a significant role in these diseases, in addition to a series of immunological phenomena which lead to the development of blisters.

Inflammatory cells are the main source of MMPs. Neutrophils forming influxes in the skin release the matrix and immunoglobulin-degrading enzymes [24, 25]. Cytokine-secreting lymphocytes are a cause of polynuclear cell influx and the activation of endopeptidases [24, 26].

Recent literature data shows that IgA-Ag complexes presented in the tissue in a course of different IgA-mediated diseases, also DH, are responsible for tissue damage due to migration induction as well as granulocyte activation. This is why granulocytes are unable to destruct the complexes which lead to attract new multinuclear cells, sustain inflammation, and, as a consequence, cause tissue damage [27].

Our findings confirming the presence of TNF*α* and MMP9 in tissues from patients with DH confirm the role of IgA-Ag complexes mentioned above. Neutrophils are one of the major sources of those substances and act as important inflammatory cells. Most importantly, they possess the Fc*α*RI surface receptor, which allows them to link to IgA and activate the tissue damage process.

The autocrine and paracrine effects of cytokines on the inflammatory cells and keratinocytes appear to cause an imbalance between MMPs and their inhibitors. This phenomenon leads to changes in the architecture of the extracellular matrix [26, 28, 29]. Some authors [29–31] have confirmed that collagenase and elastase are released mainly from inflammatory cells, regardless of the constitutive secretion of these enzymes by keratinocytes [32–34].

Previous reports confirm the increased activity of stromelysin 1 in skin lesions in patients with DH. It is an enzyme which degrades the proteins of the basement membrane and activates procolagenasis. No increased expression of other MMPs such as matrilysin has been observed, although they may potentially cause proteolysis of basement membrane proteins such as entactin and type IV collagen [35]. In our study, MMP9 expression was found not only in basal keratinocytes but also in all other layers of the epidermis in patients with BP. Metalloproteinase 9 probably contributes to the formation of blisters by the degradation of the anchoring fibres [34].

Our experiences confirm those reports. The high expression of MMP9 was observed in basal keratinocytes, in areas of intense neutrophilic infiltration, and in blister fluid. However, the results show the possibility of stimulating the production of metalloproteinases by other layers of the epidermis. MMP9 is probably the primary element responsible for the formation of lesions in the course of BP [32]. Fluid extracted from the newly formed blister may be a good material for the assessment of the activity of metalloproteinase in skin lesions.

Recent reports suggest that collagen XVII and collagen VII, which are the basis of anchoring fibers, undergo proteolysis under the influence of a specific group of metalloproteinases. In murine models of bullous pemphigoid, Verraes et al. [34] assess the participation of neutrophil elastase and MMP9 in the formation of blisters through the proteolytic degradation of the BP antigen 180. Both of these enzymes have been found to be present in specimens from both lesions and blister fluid [34]. The results of these studies have not yet been confirmed in* in vivo* experiments, where the main infiltration cells, eosinophils, do not have the ability to secrete this type of metalloproteinase.

Based on the obtained results, it can be concluded that the increased activity of matrix metalloproteinase 9 and the consequent imbalance between the groups of enzymes are responsible for tissue destruction in the course of BP. Considering the increasing number of studies concerning the participation of MMPs in the pathology of skin diseases, research regarding the therapeutic use of MMP inhibitors can be expected in the near future.

The findings of the present study demonstrate increased expression of TNF*α* in skin lesions, both in the case of bullous pemphigoid and in dermatitis herpetiformis. This can activate the production of other inflammatory mediators. Elevated PAF and MMP9 levels in the sera of patients may indicate the activation of mast cells in the process of blister formation in these diseases. In the present study, we investigated the relative contribution of mediators of mast cells in the immunopathogenesis of BP and DH skin lesions. We conclude that mast cells are active participants in events that mediate tissue damage in autoimmune disease. Disease-associated increases in mast cell numbers accompanied by mast cell degranulation and elaboration of numerous mast cell mediators at sites of inflammation are commonly observed in many human autoimmune diseases, such as multiple sclerosis and rheumatoid arthritis. In animal models, treatment with mast cell stabilizing drugs or mast cell ablation can result in diminished disease [35].

## Figures and Tables

**Table 1:** The quantitative data of TNF*α* and MMP9 positive cells expressed in BP, DH, and control group (L), in lesion (S) and in the surrounding skin.

TNF*α*	DH	BP	Control
L			
Mean	1.39	1.59	0.16
SEM	0.069	0.100	0.017
Median	1.38	1.48	0.15
Range	0.50	1.52	0.20

S			
Mean	0.62	1.01	0.16
SEM	0.050	0.045	0.017
Median	0.64	0.94	0.15
Range	0.35	0.87	0.20

MMP9	DH	BP	Control

L			
Mean	2.34	7.41	2.15
SEM	0.356	0.814	0.276
Median	2.51	7.66	2.68
Range	2.52	14.57	2.63

S			
Mean	1.69	2.01	2.15
SEM	0.511	0.183	0.276
Median	1.09	1.81	2.68
Range	3.64	3.76	2.63
